# Comparison of gemcitabine and anthracycline antibiotics in prevention of superficial bladder cancer recurrence

**DOI:** 10.1186/s12894-019-0530-0

**Published:** 2019-10-15

**Authors:** Tian-Wei Wang, Hui Yuan, Wen-Li Diao, Rong Yang, Xiao-Zhi Zhao, Hong-Qian Guo

**Affiliations:** 1 Nanjing Medical University, 101 Longmian Rd, Nanjing, 211166 China; 2 Department of Urology, Nanjing Drum Tower Hospital, Medical School of Nanjing University, 321 Zhongshan Rd., Nanjing, 210008 China

**Keywords:** Gemcitabine, Anthracycline antibiotics, NMIBC, Intravesical therapy

## Abstract

**Background:**

Because of the failure, shortage and related toxicities of Bacillus Calmette-Guérin (BCG), the other intravesical chemotherapy drugs are also widely used in clinical application. Gemcitabine and anthracycline antibiotics (epirubicin and pirarubicin) are widely used as first-line or salvage therapy, but which drug is better is less discussed.

**Methods:**

A total of 124 primary NMIBC patients administered intravesical therapy after transurethral resection of bladder tumor (TURBT) at Nanjing Drum Tower hospital from January 1996 to July 2018. After TURBT, all patients accepted standard intravesical chemotherapy. Recurrence was defined as the occurrence of a new tumor in the bladder. Progression was defined as confirmed tumor invading muscular layer. Treatment failure was defined as need for radical cystectomy (RC), systemic chemotherapy and radiation therapy.

**Results:**

Of the 124 patients who underwent intravesical chemotherapy, 84 patients were given gemcitabine, 40 patients were given epirubicin or pirarubicin, with mean follow-up times (mean ± SD) of (34.8 ± 17.9) and (35.9 ± 22.1) months respectively. The clinical and pathological features of patients show no difference between two groups. Recurrence rate of patients given gemcitabine was 8.33% (7 out of 84), the recurrence rate was 45% (18 out of 40) for epirubicin or pirarubicin (*P* < 0.0001). The progression rates of gemcitabine, anthracycline antibiotics groups were 2.38% (2 out of 84) and 20% (8 out of 40), respectively (*P* < 0.001). The rate of treatment failure is 8.33% (7 out of 84) and 25% (10 out of 40), respectively (*P* = 0.012). Gemcitabine intravesical chemotherapy group was significantly related to a lower rate of recurrence (HR = 0.165, 95% CI 0.069–0.397, *P* = 0.000), progression (HR = 0.160, 95% CI 0.032–0.799, *P* = 0.026) and treatment failure (HR = 0.260, 95% CI 0.078–0.867, *P* = 0.028).

**Conclusion:**

In conclusion, gemcitabine intravesical chemotherapy group was significantly related to a lower rate of recurrence, progression and treatment failure. Gemcitabine could be considered as a choice for these patients who are not suitable for BCG.

## Background

According to EAU Guidelines, bladder cancer (BCa) is the 11th mostly diagnosed cancer in the population. About 75% of bladder cancers are NMIBC at initial diagnosis [1], 60% of these patients experience recurrence and 10% experience progression in 5 years [2].T1 tumor, HG/G3tumor, CIS, multifocal, recurrent before and tumor size is larger than 3 cm are regarded as high-risk tumors. Micropapillary, plasmocytoid, nested, sarcomatoid, microcystic, squamous, and adeno variants of urothelial carcinoma have a poor prognosis [1]. In order to control disease recurrence and progression, intravesical therapy is conventional used after TURBT.

Intravesical BCG therapy is a standard treatment for NMIBC after TURBT [3]. Although BCG has been regarded as the most effective intravesical therapy, it also has disadvantages in clinical use. Firstly, intravesical BCG therapy is associated with adverse effects such as reactive arthritis [4] and Poncet’s disease [5]. Secondly, the production of BCG can’t meet the market demand, which leads to a world-wide shortage of BCG [6]. Thirdly, up to 40% patients do not respond to intravesical BCG therapy [7]. For all these reasons and more, gemcitabine and anthracycline antibiotics are also widely used clinically as first-line therapy or salvage therapy. However, whether gemcitabine is superior to other intravesical chemotherapy drugs is rarely discussed.

In the present study, we aimed to assess the impact of different intravesical chemotherapy drugs on recurrence, progression and treatment failure in patients with NMIBC.

## Methods

A total of 124 primary NMIBC patients administered intravesical therapy after TURBT at Nanjing Drum Tower hospital from January 1996 to July 2018 were retrospectively analyzed. Inclusion criteria: The patients were primary diagnosed with NMIBC and all of them accepted TURBT followed by intravesical therapy; The demographic, clinical and pathological information was accurate. Histology was affirmed by experienced pathologists at the department of pathology. The grade classification of urothelium carcinomas was according to 2004 WHO classifications and the TNM classification was based on 2002 TNM classification approved by the Union Internationale Contre le Cancer. All patients were stratified according to AUA risk strata. The surgeons evaluated the location, size and numbers of tumors during the operations. All of these patients accepted the immediate chemotherapy after operation. The intravesical therapeutic regimen is shown below: Perfusion once a week for 6 weeks; then once every 2 weeks for 12 weeks; next once a month for 6 months; following once every 2 months until a full year. The respectively per dosage of gemcitabine, pirarubicin and epirubicin is 1000 mg, 40 mg and 40 mg. Treatment plan was timely adjusted according to the review results.

### Follow up

Patients were followed up every 3 months with urine cytology and cystoscopy during the first year, every 6 months for the next 2 years, and then yearly thereafter. Ultrasonography, CT scanning, cystoscopy and urinary cytology were used to evaluate recurrence. Recurrence was defined as the occurrence of a new tumor in the bladder. Progression was defined as confirmed tumor invading muscular layer. Treatment failure was defined as need for radical cystectomy (RC), systemic chemotherapy and radiation therapy.

### Statistical analysis

Statistical analysis was performed using SPSS version 22.0. The categorized data was presented as count value, the continuous variables were reported as mean ± SD. The continuous and categorized data were compared using the t test and Chi square test. Meier method was used to generate the survival curves. The log-rank test was used to verify statistical significance between curves. The multivariable proportional hazards model was used to test prognostic factors with *P* < 0.2 in univariate analysis. Multivariate analyses of data were performed using the Cox proportional hazards model. Figures drawing were performing with GraphPad Prism 7. For all statistical comparisons, a value of *p* < 0.05 was considered statistically significant.

## Results

Of the 124 patients who underwent intravesical chemotherapy at Nanjing drum tower hospital, 84 patients were given gemcitabine, 40 patients were given epirubicin or pirarubicin, with mean follow-up times (mean ± SD) of (34.8 ± 17.9) and (35.9 ± 22.1) months respectively. The clinical and pathological features of patients show no difference between two groups. The baseline characteristics of patients according to treatments are shown in Table 1.

**Table 1:** Characteristics of patients

Patient data	Intravesical chemotherapy drug		*P*
	Gemcitabine (*n* = 84) *n*(%)	Epirubicin or Pirarubicin (*n* = 40) *n*(%)	
Gender			
Male	61 (72.62%)	30 (75%)	0.779
Female	23 (27.38%)	10 (25%)	
Age (years)			
< 65	39 (46.43%)	14 (35%)	0.229
≥ 65	45 (53.57%)	26 (65%)	
Multifocality			
Single	41 (53.25%)	20 (57.14%)	0.701
Multiple	36 (46.75%)	15 (42.86%)	
Size (cm)			
< 3	57 (67.86%)	25 (62.5%)	0.427
≥ 3	27 (32.14%)	15 (37.5%)	
Grade			
Low	42 (57.53%)	19 (51.35%)	0.538
High	31 (42.47%)	18 (48.65%)	
Risk			
Low	19 (22.62%)	13 (32.50%)	0.452
Immediate	25 (29.76%)	9 (22.50%)	
High	40 (47.62%)	18 (45.00%)	
reTURBT			
Yes	33 (39.29%)	17 (42.50%)	0.733
No	51 (60.71%)	23 (57.50%)	
Follow up months			
(mean ± SD)	34.8 (17.9)	35.9 (22.1)	0.772

Clinical outcome of the two groups is shown in Table 2. Recurrence rate of patients who was given gemcitabine was 8.33% (7 out of 84), the recurrence rates were 45% (18 out of 40) for epirubicin or pirarubicin (*P* < 0.001). The progression rates of gemcitabine, epirubicin or pirarubicin groups were 2.38% (2 out of 84) and 20% (8 out of 40), respectively (*P* < 0.01). The rate of treatment failure was 8.33% (7 out of 84) and 25% (10 out of 40), respectively (*P* < 0.05), as shown in Table 2. Taken together, intravesical chemotherapy with different drugs showed an obvious statistically difference, the epirubicin or pirarubicin group had a higher recurrence free survival, progression free survival and treatment failure free survival rate than gemcitabine group.

**Table 2:** Recurrence, progression and treatment failure rates of the two groups

	Gemcitabine	Epirubicin or Pirarubicin	*P*
Recurrence			
Yes	7 (8.33%)	18 (45%)	0.000
No	77 (91.67%)	22 (55%)	
Progression			
Yes	2 (2.38%)	8 (20%)	0.001
No	82 (97.62%)	32 (80%)	
Treatment failure			
Yes	7 (8.33%)	10 (25%)	0.012
No	77 (91.67%)	30 (75%)	

**Fig. 1:**
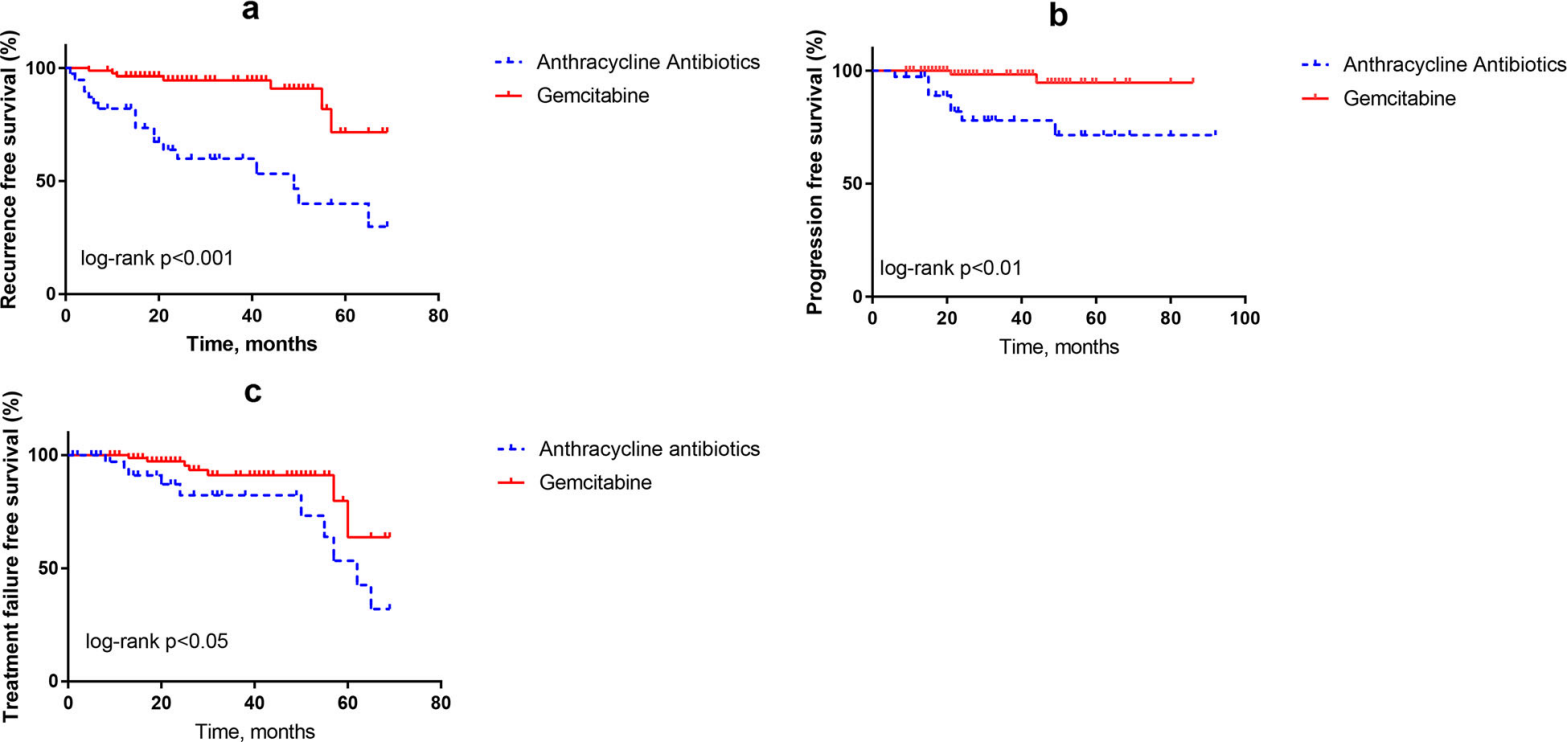
**a**-**c** Kaplan–Meier curves for all patients. **a** Recurrence free survival; **b** progression free survival; **c** Treatment failure free survival. Tumor recurrence, progression and treatment failure differ significantly between these groups. Comparing with epirubin or pirarubicin group, gemcitabine group showed obvious advantage in inhibiting tumor recurrence, progression and treatment failure

The log-rank tests show obvious differences between the two groups in recurrence free survival rates, progression free survival rates and treatment failure free survival rates. The Kaplan-Meier estimates of two groups are graphically presented in Fig. 1a–c. Tumor recurrence, progression and treatment failure differ significantly between these groups. Comparing with epirubin or pirarubicin group, gemcitabine group showed obvious advantage in inhibiting tumor recurrence, progression and treatment failure. After univariate analysis, variables with *p* < 0.2 were enrolled into multivariate analysis (Table 3). Gemcitabine intravesical chemotherapy group was significantly related to a lower rate of recurrence (HR = 0.165, 95% CI 0.069–0.397, *P* = 0.000), lower rate of progression (HR = 0.160, 95% CI 0.032–0.799, *P* = 0.026) and treatment failure (HR = 0.260, 95% CI 0.078–0.867, *P* = 0.028).

**Table 3:** Univariate and multivariate standard Cox proportional hazards analysis of variables associated with tumor recurrence, progression and treatment failure in all patients

Variable	Univariate HR (95% CI)	*P*	Multivariate HR (95% CI)	*p*
Recurrence				
Gender	1.338 (0.817–2.191)	0.247		
Age	1.497 (0.658–3.403)	0.336		
Multifocality	1.526 (0.642–3.629)	0.339		
Size	1.869 (0.850–4.108)	0.120	2.668 (1.146–6.215)	0.023
Grade	0.443 (0.190–1.033)	0.060	1.243 (0.232–6.664)	0.800
Risk				
Low^a^				
Immediate	1.934 (0.696–5.371)	0.206	2.762 (0.461–16.543)	0.266
High	3.019 (1.186–7.685)	0.020	3.632 (0.535–24.633)	0.187
reTURBT	0.441 (0.182–1.068)	0.070	0.667 (0.134–3.313)	0.620
Gemcitabine vs Epirubicin or Pirarubicin	0.159 (0.066–0.382)	0.000	0.179 (0.072–0.447)	< 0.001
Progression				
Gender	1.266 (0.583–2.747)	0.552		
Age	0.765 (0.221–2.644)	0.672		
Multifocality	4.063 (0.819–20.143)	0.086	4.168 (0.827–21.000)	0.084
Size	2.188 (0.195–24.519)	0.525		
Grade	1.333 (0.376–4.727)	0.657		
Risk				
Low^a^				
Immediate	0.993 (0.181–5.432)	0.993		
High	1.945 (0.486–7.780)	0.347		
reTURBT	0.323 (0.068–1.521)	0.153	0.278 (0.055–1.394)	0.120
Gemcitabine vs Epirubicin or Pirarubicin	0.110 (0.023–0.518)	0.005	0.155 (0.031–0.781)	0.024
Treatment failure				
Gender	0.447 (0.128–1.561)	0.207		
Age	2.094 (0.736–5.960)	0.166	2.244 (0.591–8.527)	0.235
Multifocality	2.156 (0.739–6.290)	0.160	1.447 (0.479–4.368)	0.512
Size	0.897 (0.314–2.562)	0.840		
Grade	1.485 (0.491–4.485)	0.484		
Risk				
Low^a^				
Immediate	1.315 (0.443–3.906)	0.622		
High	0.455 (0.099–2.080)	0.310		
reTURBT	1.429 (0.537–3.804)	0.475		
Gemcitabine vs Epirubicin or Pirarubicin	0.377 (0.140–1.018)	0.054	0.248 (0.074–0.830)	0.024

^a^Referenced category. reTURBT, repeated transurethral resection of bladder tumor

### Toxicity evaluation

Common side effects in both groups included chemical cystitis, urinary frequency, hematuria and suprapubic discomfort. Overall 4% of patients experienced side effects and no patients stopped chemotherapy because of side effects. None of the toxicities were fatal.

## Discussion

Although BCG is used as first-line intravesical therapy for NMIBC patients after TURBT, its shortcomings also poses a management dilemma in clinical application, which force clinicians to search for better therapeutic strategies. As some patients do not respond to BCG or cannot tolerate its side effects, Yang et al. used Gemcitabine and cisplatin (GC) adjuvant chemotherapy instead of BCG intravesical therapy, which showed favorable results [8]. Some researches tried to reduce the standard dose of BCG to lessen the shortage of BCG. However, there is no consensus suggesting that intravesical BCG standard dose can be replaced by now [9]. Kyla N. Velaer et al. reported their experience on sequential intravesical gemcitabine and docetaxel as salvage therapy after BCG failure [10]. With the growth demand of BCG and the increasing number of patients, the contradiction will become more and more prominent.

Due to various reasons, gemcitabine is widely used in bladder cancer. In Australia, gemcitabine was setted as first-line intravesical therapy since 2010 [11]. Thiru Prasanna et al. deemed that intravesical gemcitabine had better DFS and lower toxicity when compared with BCG [11]. Pirarubicin and epirubicin has been widely used in intravesical chemotherapy since 1980s and has been proved to be effective. However, William B. Tabayoyong et al. collected seven trials about epirubicin and reported that six trials showed no improvement in recurrence with the maintenance treatment to induction [12]. What’s more, most researchers think pirarubicin and epirubicin have been only able to reduce recurrence but not progression [13]. Gemcitabine, Pirarubicin and epirubicin are widely used in China, but seldom assessed the efficiency between these therapeutic choices.

In our research, we found a trend toward better recurrence free survival, progression free survival and treatment failure free survival in gemcitabine group. It is also important that gemcitabine intravesical chemotherapy is an independent protective factor not only for recurrence, but also for progression and treatment failure. Through multivariate analysis, we noted that the size of tumor larger than 3 cm is more likely to recurrent, which is in accord with EAU Guidelines.

There are potential limitations in our analysis. First, it is a retrospective analysis, and our material have limited data. Second, the current study never includes BCG treatment because of limited data. Third, there were other variables never bring into consideration such as molecular subtype of urothelial carcinoma, which may have influenced on results. Most importantly, the limited data may makes the estimate of the treatment effect less robust. Furthermore, a large randomized controlled trial is required to clarify the importance of gemcitabine therapy.

## Conclusion

In conclusion, gemcitabine intravesical chemotherapy group was significantly related to a lower rate of recurrence, progression and treatment failure compared to anthracycline antibiotics group. Gemcitabine is superior to epirubicin or pirarubicin in inhibiting tumor recurrence and progression. We deduce that gemcitabine is also better than epirubicin or pirarubicin in salvage therapy and we will further discuss it. BCG is still the first-line intravesical therapy, but gemcitabine could be considered as a choice for those patients who are not suitable for BCG.

## Data Availability

The datasets used during the current study are available from the corresponding author or first author on reasonable request.

## Abbreviations

AUA: American urological association
BCa: Bladder cancer
BCG: Bacillus Calmette-Guérin
DFS: Disease free survival
EAU: European Association of Urology
GC: Gemcitabine and cisplatin adjuvant chemotherapy
NMIBC: Non-muscle invasive bladder cancer
RC: Radical cystectomy
TURBT: Transurethral resection of bladder tumor
